# Patterns of girdle shape and their correlates in Australian limb-reduced skinks

**DOI:** 10.1098/rspb.2024.1653

**Published:** 2024-10-02

**Authors:** Marco Camaiti, Mark N. Hutchinson, Christy A. Hipsley, Rocio Aguilar, Jay Black, David G. Chapple, Alistair R. Evans

**Affiliations:** ^1^ School of Biological Sciences, Monash University, Clayton, Victoria, Australia; ^2^ Department of Life Sciences, Natural History Museum, London SW7 5BD, UK; ^3^ South Australian Museum, Adelaide, South Australia, Australia; ^4^ School of Biological Sciences, University of Adelaide, Adelaide, South Australia, Australia; ^5^ Faculty of Science and Engineering, Flinders University of South Australia, Bedford Park, South Australia, Australia; ^6^ Department of Biology, University of Copenhagen, Copenhagen, Denmark; ^7^ Department of Sciences, Museums Victoria, Carlton, Victoria, Australia; ^8^ School of Earth Sciences, University of Melbourne, Carlton, Victoria, Australia

**Keywords:** limb reduction, squamates, Scincidae, girdles, locomotion, development

## Abstract

The evolution of limb reduction in squamates is a classic example of convergence, but the skeletal morphological patterns associated with it are underexplored. To provide insights on the biomechanical and developmental consequences of transitions to limb reduction, we use geometric morphometrics to examine the morphology of pectoral and pelvic girdles in 90 species of limb-reduced skinks and their fully limbed relatives. Clavicle shapes converge towards an acute anterior bend when forelimbs are lost but hindlimbs are retained—a morphology typical of sand-swimmers. This may either indicate functional adaptations to locomotion in fine substrates, or a developmental consequence of complete limb loss. The shape of limb-bearing elements of both girdles (coracoid and pelvis) instead closely mirrors limb reduction, becoming more simplified as undulation replaces limbed locomotion. Integration between girdles decreases in taxa lacking elements of the forelimbs but not hindlimbs, indicating differential selection on each girdle in response to distinct locomotory strategies. However, this pattern becomes less clear when considering phylogenetic history, perhaps because it is limited to one specific clade (*Lerista*). We show how the functional demands of locomotion can induce changes at different levels of organismal organization, including both external and internal structures.

## 1. Introduction

Reduction of tetrapod limbs is marked by the simplification or loss of elements of the appendicular system, including bones, cartilage and muscle [1–5]. As the repeated evolution of vertebrate limb reduction represents a convenient opportunity to study complex macroevolutionary transitions in morphology—especially in lineages where it has happened often, such as squamate reptiles—several studies [6–10] have explored evolutionary shifts in the appendicular apparatus. This system, however, does not solely comprise limbs. Pectoral and pelvic girdles are elements anchoring paired appendages to the vertebral column, acting as boundary regions between appendicular and axial domains. From this position, the girdles transmit forces involved in limbed locomotion between the two domains and absorb mechanical stresses [11].

But what changes do girdles undergo when limbed locomotion becomes less prominent? As undulating, axial-based locomotion gradually reduces reliance on limbs, these undergo reduction and loss of elements, and the axial system becomes more elongated [12–14] (but not less regionalized [15]). The structures linking these domains are also affected, as they are ontogenetically entangled with the limbs, sharing a similar developmental origin [16], and must accommodate biomechanical shifts in bone placement and muscle attachments [11,12]. Reductions and losses in girdle elements in relation to limb reduction are well documented across squamate lineages, having been investigated in amphisbaenians [4,17], snakes [18,19], skinks [16,20–23], pygopodids [24] and gymnophthalmids [3,25–27].

Despite this, other than a single study [21], the shapes of the girdles of limb-reduced squamates have not been quantified beyond qualitative descriptions. Moreover, associations between girdle shapes and the patterns of limb reduction have not been examined beyond the genus level or compared across lineages in a phylogenetic context. As body shape varies substantially across and within clades, following independent and sometimes unique trajectories [9,10], an unresolved question is how diversity in limb proportions translates to variation in girdle morphology. Mapping variation in girdle shape and loss of limb bone elements across a wide range of taxa with varying degrees of limb reduction would provide a finer understanding of the evolutionary pathways of various groups of limb-reduced and limbless reptiles. This would discriminate between imperfect morphological convergence, referring to distinct evolutionary trajectories in response to common adaptive regimes leading to similar but not identical outcomes [28,29], and true convergence, where the processes that lead to given morphologies are repeated across clades [9].

Another key unresolved question relates to the functional interactions between skeletal morphology, body proportions and ecology. While patterns of body shape variation have been linked to differences in locomotor mode and ecomorphology in limb-reduced lineages (Grizante *et al*. [30] for gymnophthalmids; Camaiti *et al.* [10], Morinaga & Bergmann [31] for skinks), it remains unexplored how these differences translate to shifts in musculoskeletal morphology. As highlighted by studies on other squamates such as anoles [32–36], habitat specialization may require the evolution of different musculoskeletal adaptations to make locomotory performance more efficient. Given that limb reduction and trunk elongation are thought to evolve to improve the efficiency of locomotion through complex three-dimensional (3D) media [10,12,37], girdles may also be modified in response to the same selective pressures. As with limb reduction, different types of substrate may drive evolutionary shifts in pectoral and pelvic girdle morphology [26].

Here, we examine variation in pectoral and pelvic girdle morphology of limb-reduced lizards, and their relationships with limb-reduced body shapes. In particular, we aim to answer the following questions:

— Do external patterns of limb reduction translate into variation at the levels of girdles, and does this demonstrate convergent patterns across clades? More specifically, how predictive is the reduction and loss of distinctive limb elements to girdle shape?
— Is variation in pectoral and pelvic girdle shape subject to the functional demands of locomotion within different substrates similar to the limbs?

To address these questions, we use geometric morphometrics to compare the 3D shapes of the elements of the pectoral and pelvic regions, and examine to which degree they covary with ecology and body shape across 90 squamate species, including limb-reduced forms and their fully limbed relatives. Our focal taxa belong to a clade that evolved limb reduction several times independently, the Australian sphenomorphine skinks (Scincidae: Lygosominae: Sphenomorphini [38,39]). Skinks in general, and Sphenomorphini in particular, exhibit a great diversity of patterns of limb reduction [40,41]. These differ in the degree to which limbs are reduced and the directionality of reduction, with species reducing forelimbs faster than hindlimbs, or *vice versa* [2,9,42].

## 2. Material and methods

### (a) Taxon sampling, data extraction and landmark protocol

Computed tomographic image stacks (microCT scans) of 90 specimens of Australian skinks were acquired using a SkyScan 1076 microCT scanner at Adelaide Microscopy (University of Adelaide, Adelaide, South Australia, Australia) and a Phoenix Nanotom at the University of Melbourne School of Geography, Earth and Atmospheric Sciences TrACEES Platform (University of Melbourne, Melbourne, Victoria, Australia).

Specimens and specimen numbers used in this study are provided in electronic supplementary material S1A. Each specimen represents a skink species from the Australian branch of the Tribe Sphenomorphini [39]. We sourced representatives of limb-reduced clades belonging to this group, including the genera *Anomalopus*, *Calyptotis*, *Coeranoscincus*, *Coggeria*, *Glaphyromorphus*, *Hemiergis*, *Lerista*, *Ophioscincus* and *Saiphos*, adding allied fully limbed genera *Ctenotus*, *Concinnia*, *Eremiascincus*, *Eulamprus* and *Gnypetoscincus*, to inform morphological comparisons (like Camaiti *et al.* [10]).

Avizo (v. 2022.2, Thermo Fisher Scientific) was used to visualize 3D volumes from reconstructed stacks. The same was used to digitally isolate the pectoral and pelvic girdles of each specimen, which were then exported as 3D ‘.ply’ meshes. When either region was damaged (in seven specimens: electronic supplementary material S1A), meshes were repaired and realigned using Geomagic Wrap (v. 2021) on a case-to-case basis [43] with respect to the recognizable anatomy of each specimen, attempting to maintain symmetry.

We placed landmarks and semilandmarks on meshes using IDAV Landmark Editor (v. 3.6), using our own protocol (electronic supplementary material S2). The pectoral girdle protocol bears resemblance to Koeller [21] but quantifies only clavicle and scapulocoracoid shape. Each was analysed in isolation. One specimen was excluded (*Anomalopus verreauxii*) due to deformation of both clavicles. We placed a curve of 46 sliding semilandmarks on the right clavicle, starting at the acromion process [11], continuing along the anterior edge of the clavicle as it curves medially to meet the opposite clavicle, ending at the angle between the anteromedial edge and the posterior edge (electronic supplementary material S2A). For two specimens (electronic supplementary material S1), this protocol was applied to the left clavicle because of its better preservation, placing a curve starting laterally and ending medially; landmark configuration for these specimens was mirrored by multiplying one landmark dimension by −1.

The scapulocoracoid was present in all specimens except one (*Glaphyromorphus fuscicaudis*, due to an incomplete scan). We placed a curve of 28 sliding semilandmarks on the posterolateral edge of the scapulocoracoid, starting where the scapular bone meets the suprascapular, down across the edge of the glenoid fossa (or where it would be), to where the coracoid meets the epicoracoid (electronic supplementary material S2B). This was done on the right scapulocoracoid for most specimens (*n* = 75); for the remainder (*n* = 14) where the left element was better preserved, we applied the same protocol and mirrored it by multiplying one landmark dimension by −1.

Besides these curves, no additional landmarks were placed on the clavicle or scapulocoracoid, as further homologous structures could not be universally parsed from our dataset.

The landmarking protocol used for the pelvic girdle (electronic supplementary material S2C) was modified from Tinius *et al*. [34] for anoles, with differences due to the inconsistent presence of pelvic processes in skinks compared with anoles (i.e. preacetabular processes). Limbless species (*n* = 3/90) were excluded as their pelvises lacked identifiable homologous points. From the right side, the first fixed landmark was placed on the posterior edge of the pubis, where it forms an angle with the contact surface with the epipubis. The second was placed on the anterior edge of the contact surface of the pubis, the third on the posterior edge of the ventral tip of the pubic apron, the fourth on the lateral protrusion of the anterior acetabular extremity, the fifth on the end of the ridge at the ventral extremity of the ilium and the sixth on the extremity of the dorsal ridge of the ilium. The seventh was placed on the posteroventral extremity of the acetabulum, the eighth on the posterior tip of the ischiatic tuberosity, the ninth on the anterolateral tip of the ischium. The protocol (landmarks 10–18) was repeated in this sequence on the other side of the pelvis (electronic supplementary material S2C). Landmark configurations of the clavicle, scapulocoracoid and pelvic girdle are provided in electronic supplementary material S2A–C, respectively.

To explore associations between girdle morphology and body shape, we sourced morphometric and count measurements (head length, snout-vent length (SVL), limb lengths, digit numbers, number of presacral vertebrae, number of pairs of limbs) from the datasets of Camaiti *et al.* [10,42]. For species absent from those datasets (*Calyptotis lepidorostrum*, *Concinnia tenuis*, *Eulamprus kosciuskoi*, *Glaphyromorphus cracens*), we took measurements from meshes via the line measuring tool in Avizo (v.2022) and counted presacral vertebrae and digits. We calculated limb disparity, relative limb lengths, and trunk elongation based on Camaiti *et al.*’s [10] definitions and methods. Limb status—whether a species is fully limbed, limb-reduced or limbless—was assigned to each species based on the definition by Camaiti *et al.* [42], or lacking that, on measurements obtained from scans (electronic supplementary material S3). By that definition, ‘limb-reduced’ species have forelimb lengths ≤15%, and/or hindlimb lengths ≤20% of SVL, and ‘limbless’ species have no external limbs. To further distinguish between degrees of limb reduction, limb-reduced species were split between ‘highly reduced’, when at least one of the limb pairs was ≤5% of SVL, and ‘moderately reduced’ (all remaining species). We also counted the presence and number of the sequential bones of the limbs from 3D scans, creating an ordered scale of expression of osteological elements for each limb (‘Forelimb expression’ (FE) and ‘Hindlimb expression’ (HE) for the respective limbs). This was scored as follows: (0) absence of limb elements; (1) only stylopodium (humerus or femur) present; (2) only stylopodium and zeugopodium (radius-ulna or tibia-fibula) present; (3) from the presence of any podial bone up to three phalanges in the digit with the highest number of phalanges; (4) four phalanges in the digit with the highest number of phalanges; (5) five phalanges in the digit with the highest number of phalanges. This parameter describes the number of sequentially ossified units that constitute the limbs (electronic supplementary material S1E).

We classified species based on the substrate ecology categories of Camaiti *et al.* [10,37,42], assigning categories (sand, sandy soil, soil, humus, surface-dweller) to species missing from that dataset based on the literature (electronic supplementary material S3).

### (b) Data analysis

Landmark configurations were separately exported and aligned via Procrustes superimposition via the ‘gpagen’ function of *geomorph* (v. 3.3.1: [44]) in R, specifying fixed and sliding semilandmarks. For phylogenetic corrections, we used the phylogeny of Zheng & Wiens [45], using the ‘treedata’ function to match our species to the tips of the phylogenetic tree and prune other species in the phylogeny (package *geiger*, v. 2.0.7: [46]). We estimated multivariate phylogenetic signal *K* (*K*_mult_: [47]) of pectoral and pelvic girdle shapes via the ‘physignal’ function in *geomorph* [44]. We include considerations of the effects of asymmetry on our assessment of pelvic shape in electronic supplementary material S2E.

We used the ‘gm.prcomp’ function to quantify and visualize pelvic, clavicular and scapulocoracoid shape in principal component analyses (PCAs) (in *geomorph* [44]), and the ‘phylomorphospace’ function (*phytools*, v. 0.7.7: [48]) to visualize phylogenetic relationships across the morphospace. To assess the relationship between the shape of clavicle, scapulocoracoid and pelvic girdle, we used the function ‘two.b.pls’ for each combination, the ‘phylo.integration’ function to visualize these relationships with phylogenetic control and the function ‘compare.pls’ to compare the degree of integration among these blocks (*geomorph* [44]).

Effects of body shape variables, limb development and ecological predictors on the shapes of girdles were assessed by conducting phylogenetically corrected Procrustes analyses of variance (ANOVA), employing the ‘procD.pgls’ function in *geomorph* [44]. We used the coefficient of determination (*R* ^2^) to compare associations. For predictors with discrete character states, we performed pairwise comparisons between the states of each trait using the ‘pairwise’ function in the package *RRPP* (v. 4.0: [49]) to assess differences between states.

## 3. Results

### (a) Pectoral girdle

#### (i) Clavicle

**Figure 1.**
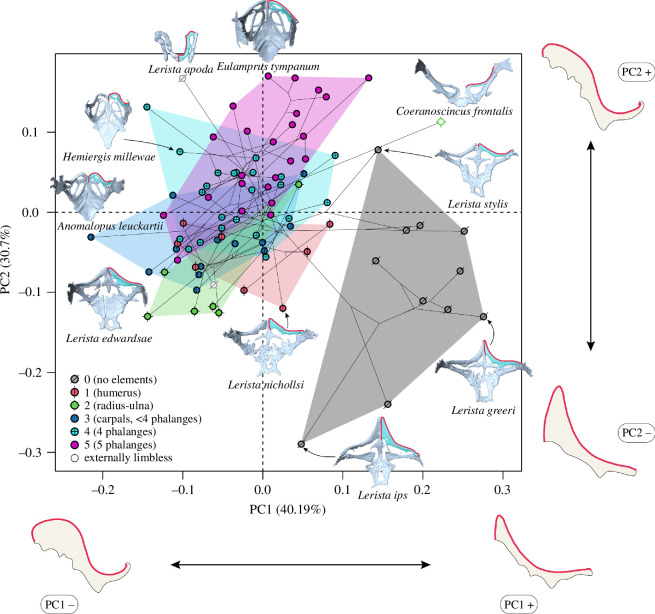
Phylomorphospace plots of the first two PC axes of clavicle shape variation. Proportions of variation explained by axes in brackets. Specimens are represented by a point, coloured by forelimb expression (FE), with white points representing species lacking all trace of external limbs. They are bounded by convex hulls coloured by character state. In red, next to each axis are shown the maximum shape deformations indicating the extremes of the variation for that axis, in dorsal view. To aid visualization, semilandmark curves are overlaid on top of illustrations of the right clavicle. Examples of pectoral girdles from our sample are included, in dorsal view, next to their position in the morphospace, with the right clavicle highlighted in a lighter colour.

PC1 (40.19% of shape variation) correlates positively with the acuteness of the anterior bend of each clavicle, and PC2 (30.7%) positively with the apex of the bend being more laterally shifted (as opposed to medially) (figure 1). The morphospace shows some phylogenetic patterns, especially on PC2, with part of the *Lerista* clade (those species lacking forelimb elements) occupying the negative region of that axis, while other clades are limited to the positive region. The shape of the clavicular margin displays a significant phylogenetic signal (*K*_mult_ = 0.61, *p* = 0.001).

Clavicle shape regressions are summarized in table 1. We retrieve no significant associations between clavicular shape and limb status. The number of limb pairs has a small effect on clavicle shape (*R* ^2^ = 0.08, *p* = 0.003). FE correlates with clavicular shape (*R* ^2^ = 0.1, *p* = 0.018), with pairwise comparisons showing a distinction between species lacking limb elements (score 0) and species having one or more elements (scores 1 to 5) (electronic supplementary material S3A). As shown both visually (figure 1) and from pairwise comparisons among FE states (electronic supplementary material S4A), all character states overlap across the morphospace. However, taxa lacking forelimb elements form a distinct cluster in the positive region of PC1, including three species groups within *Lerista* (figure 1). This distinction in morphospace occupation is not evident when considering all species with this character state, but it becomes clear when excluding limbless outliers, leaving species retaining only hindlimb elements (figure 1). HE does not correlate with clavicle shape.

**Table 1.** Results of regressions between the shapes of girdle elements and predictors of body morphology and ecology.*R* ^2^: *R*-squared; *F*: *F*-statistics; *Z*: *Z*-statistics; *P*: *p*‐value. Significant *p*-values are italicized.

	clavicle				scapulocoracoid				pelvis			
predictor ↓	*R* ^2^	*F*	*Z*	*P*	*R* ^2^	*F*	*Z*	*P*	*R* ^2^	*F*	*Z*	*P*
limb status	0.06	1.80	1.56	0.061	0.09	2.74	2.46	*0.009*	0.17	8.43	5.38	*0.001*
substrate type	0.12	2.89	3.14	*0.001*	0.06	1.41	1.05	0.145	0.06	1.31	1.04	0.155
number of limb pairs	0.08	3.88	2.67	*0.003*	0.12	6.09	3.49	*0.001*	0.02	2.07	1.44	0.092
number of fingers	0.08	1.41	1.15	0.128	0.10	1.84	*1.93*	*0.021*	0.17	3.30	4.41	*0.001*
number of toes	0.09	1.68	1.59	0.059	0.08	1.43	1.13	0.136	0.23	4.75	5.52	*0.001*
forelimb expression	0.10	1.86	*2.05*	*0.018*	0.11	2.06	2.24	*0.015*	0.21	4.22	5.32	*0.001*
hindlimb expression	0.10	1.81	1.66	0.059	0.11	1.97	1.92	*0.034*	0.15	4.80	4.33	*0.001*
relative forelimb length	0.03	2.84	1.86	*0.032*	0.07	7.02	3.19	*0.001*	0.04	3.94	*2.53*	*0.01*
relative hindlimb length	0.04	3.16	*1.83*	*0.033*	0.05	4.31	2.18	*0.017*	0.14	13.67	5.00	*0.001*
limb disparity	0.04	3.77	2.26	*0.007*	0.03	2.65	1.87	*0.04*	0.06	5.06	3.21	*0.002*
snout-vent length	0.01	0.99	0.33	0.387	0.01	1.22	0.65	0.248	0.04	3.12	2.29	*0.011*
trunk elongation	0.01	1.24	0.62	0.278	0.02	1.98	1.31	0.102	0.12	11.58	4.69	*0.001*
presacral vertebrae number	0.02	1.59	1.00	0.164	0.03	2.96	1.87	*0.029*	0.10	9.24	4.02	*0.001*

Clavicle shape correlates weakly with relative forelimb length (*R* ^2^ = 0.03, *p* = 0.032) and relative hindlimb length (*R* ^2^ = 0.035, *p* = 0.033). Limb disparity (difference in size between fore- and hindlimbs) does not correlate with clavicle shape (*R* ^2^ = 0.04, *p* = 0.07). No relationship is found between clavicle shape and SVL, trunk elongation or presacral vertebrae number. We find a significant association with substrate categories for clavicle shape (*R* ^2^ = 0.12, *p* = 0.001). Pairwise comparisons show that clavicle shape significantly differs between species from sand and sandy soil, and between soil-dwelling and surface-dwelling species (table 1).

#### (ii) Scapulocoracoid

**Figure 2.**
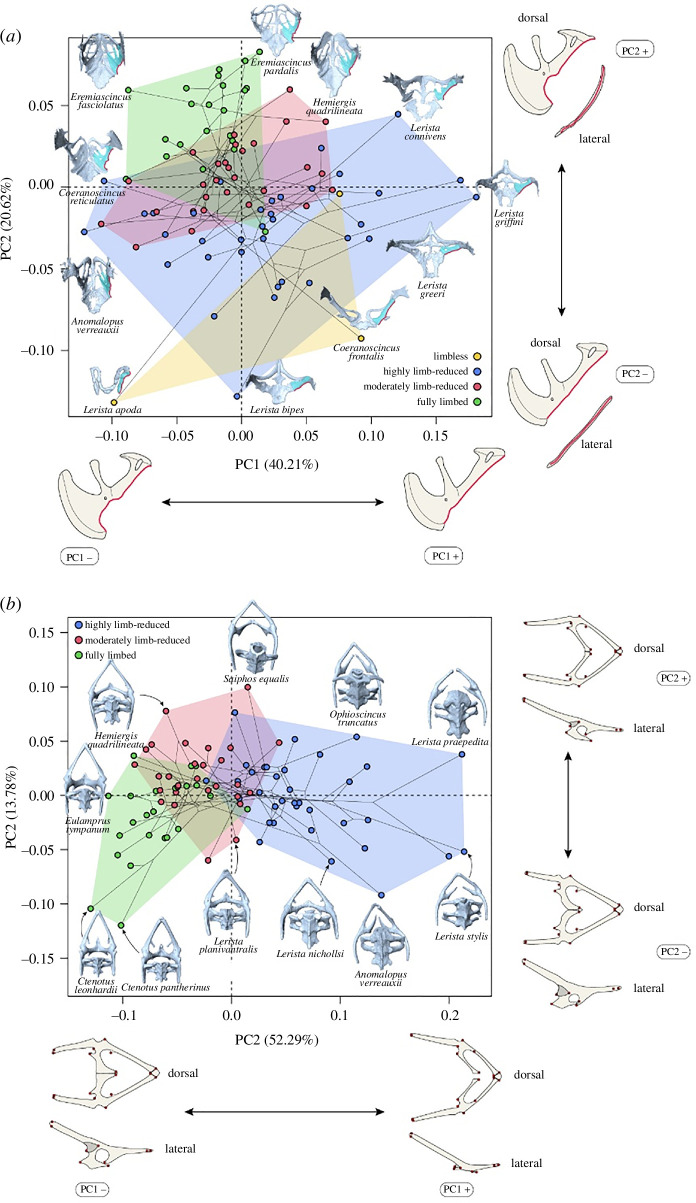
Phylomorphospace plot of the first two PC axes of (*a*) scapulocoracoid and (*b*) pelvic girdle shape variation. Proportions of shape variation explained by axes are in brackets. In both plots, each specimen is represented by a point, coloured by limb status, and bounded by convex hulls. In red, at both ends of each axis are shown the landmark configurations indicating the extremes of the variation, in dorsal and lateral view. To aid visualization, illustrations of the maximum shape deformations corresponding to these configurations are drawn around the landmarks. In (*a*), examples of pectoral girdles are shown next to their position in the morphospace, in dorsal view, with the right scapulocoracoid highlighted in a lighter colour. In (*b*), pelvic girdles from our sample are included in the plot, in dorsal view, next to their position in the morphospace.

PC1 explains 42% of the variation in the shape of the posterolateral scapulocoracoid margin, PC2, 17.38%. PC1 correlates with the decrease in depth of the glenoid fossa, PC2 with the degree of deviation from a straight line of the posterior margin of the scapulocoracoid (in lateral and dorsal view: figure 2*a*). From the top left to the bottom right of the morphospace, the margin becomes less concave. The morphospace shows stronger phylogenetic patterns than the clavicle, with *Glaphyromorphus* being most distinct on the negative PC1. The phylogenetic signal of scapulocoracoid shape is moderate and significant (*K*_mult_ = 0.45, *p* = 0.002).

Scapulocoracoid shape regression results are summarized in table 1, showing a correlation with limb status (*R* ^2^ = 0.09, *p* = 0.009). Pairwise comparisons show that fully limbed species differ from all others, and limbless species differ from moderately limb-reduced species (electronic supplementary material S4B). Scapulocoracoid margin shape weakly correlates with number of limb pairs (*R* ^2^ = 0.12, *p* = 0.001) and number of fingers (*R* ^2^ = 0.10, *p* = 0.021), but not toes (*R* ^2^ = 0.08, *p* = 0.136). It correlates with FE (*R* ^2^ = 0.11, *p* = 0.015) and HE (*R* ^2^ = 0.11, *p* = 0.034), with relative lengths of forelimbs (*R* ^2^ = 0.07, *p* = 0.001) and hindlimbs *R* ^2^ = 0.05, *p* = 0.017), weakly with limb disparity (*R* ^2^ = 0.03, *p* = 0.04) and presacral vertebrae numbers (*R* ^2^ = 0.03, *p* = 0.029). Categories of substrate ecology do not correlate with scapulocoracoid margin shape (table 1).

### (b) Pelvic girdle

#### (i) Pelvis

PC1 explains 52.29% of pelvic shape variation, PC2, 13.78%. PC1 correlates positively with an increase in the posterior opening angle between the left and right pubis, and a dorsoventral depression of the pelvic girdle (figure 2*b*). PC2 correlates positively with a laterally wider midsection of the pelvic girdle that translates the acetabulum laterally, shifting the posterior extremes of the ilia closer together (figure 2*b*).

The categories of limb reduction form an ordered gradient in the morphospace, with most of the highly limb-reduced species occupying the positive end of PC1, fully limbed species on the negative end and moderately limb-reduced species in between (figure 2*b*). Phylogenetic patterns are most evident for this element, with *Hemiergis* and *Glaphyromorphus* clustering somewhat consistently on the negative region of PC1 and *Anomalopus* on the positive one, but not maintaining this consistency across PC2, while most other clades (especially *Lerista*) span the whole morphospace (figure 2*b*). Pelvic girdle shape carries a significant multivariate phylogenetic signal (*K*_mult_ = 0.73, *p* = 0.001).

Phylogenetic regressions with pelvic shape show significant associations with limb status (*R* ^2^ = 0.17, *p* = 0.001: table 1). Pairwise comparisons among the character states of limb status highlight a significant distinction between fully limbed, moderately limb-reduced and highly limb-reduced species (no limbless species were included: electronic supplementary material S4C). Number of limb pairs does not correlate with pelvic shape (table 1). FE correlates with pelvic shape (*R* ^2^ = 0.21, *p* = 0.001), as does HE (*R* ^2^ = 0.15, *p* = 0.001).

Relationships with relative length of forelimbs (*R* ^2^ = 0.44, *p* = 0.01) and hindlimbs (*R* ^2^ = 0.14, *p* = 0.001) are significant for pelvic shape. Limb disparity associates with pelvic shape (*R* ^2^ = 0.06, *p* = 0.002). SVL is a weak but significant predictor of pelvic shape (*R* ^2^ = 0.04, *p* = 0.011), as is trunk elongation (*R* ^2^ = 0.12, *p* = 0.001). Substrate ecology does not show significant associations with pelvic shape (table 1).

### (c) Girdle integration

**Figure 3.**
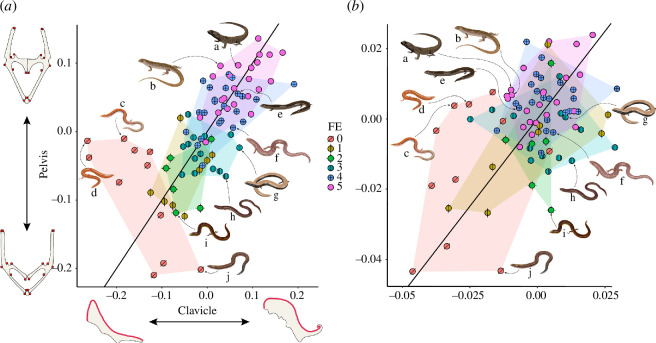
Ordination plots of (*a*) phylogenetically naive, and (*b*) phylogenetically controlled two-block partial least squares analyses between pectoral and pelvic shapes. The *x*-axis corresponds to the values of the left block (the pectoral girdle, represented by the clavicle) projected onto a singular vector; the *y*-axis corresponds to those of the right block (the pelvic girdle) projected onto a singular vector. For (*a*), at both ends of each axis are shown the landmark configurations indicating the extremes of the variation for each block, in dorsal view. Each specimen is represented by a point, coloured by forelimb expression (FE), and bounded by convex hulls. For visualization purposes, some of the species are illustrated next to their respective position in both plots, as follows: a: *Gnypetoscincus queenslandiae*; b: *Ctenotus robustus*; c: *Lerista labialis*; d: *Lerista ips*; e: *Hemiergis decresiensis*; f: *Glaphyromorphus punctulatus*; g: *Saiphos equalis*; h: *Anomalopus leuckartii*; i: *Lerista edwardsae*; j: *Lerista stylis*.

Phylogenetically naive partial least squares (PLS) analyses (figure 3*a*) retrieve a significant relationship (r-PLS = 0.64, *p* = 0.001, effect size = 7.72) between clavicle and pelvis shapes. This relationship remains significant when accounting for relatedness (figure 3*b*), albeit with a lower effect size and significance (r-PLS = 0.43, *p* = 0.024, effect size = 1.98). Scapulocoracoid shape correlates more with pelvis (r-PLS = 0.4, *p* = 0.021, effect size = 2.33) than clavicle shape (r-PLS = 0.42, *p* = 0.029, effect size = 2.24). When correcting for phylogeny, it shows significant covariance with pelvis shape (r-PLS = 0.41, *p* = 0.035, effect size = 1.92), but not with clavicle shape (r-PLS = 0.31, *p* = 0.391, effect size = 0.17). Notably, the integration with the scapulocoracoid is weaker than between clavicle and pelvis.

## 4. Discussion

### (a) Clavicle shape and the consequences of complete forelimb loss

Clavicle shape shows high degrees of overlap across categories of FE, but the clustering of taxa at the positive end of PC1 shows consistent morphological changes occurring whenever forelimb bones are completely lost, but hindlimbs are retained. This correlates with a clavicle with an acute angle in its primary curvature (figure 1). Given that this pattern occurs in three lineages (all within the genus *Lerista*), it appears to be a case of evolutionary convergence. While recent studies [9,10,50] found that different lineages evolved limb reduction by following divergent evolutionary trajectories, one [10] also highlighted how selective pressures from functional interactions with similar environments generate convergent outcomes in terms of body shape across lineages. Koeller [21] suggested that convergent clavicle morphology may be due to similar degrees of limb reduction, but our findings indicate that clavicle shape is also explained by substrate ecology, and by loss of forelimb elements (table 1). This suggests that some clavicle shapes might be actively selected by functional locomotory interactions with specific substrates. In fact, all species forming the cluster at the positive end of PC1 (figure 1) invariably inhabit either sand or sandy soil, being sand-swimmers, a strategy known to often involve hindlimb-propelled locomotion [10,51]. While these are not the only sand-swimmers in our sample, this finding shows that evolving this morphology may constitute one of many possible skeletal adaptations to improve efficiency of locomotion in granular fluids such as sand [52,53]. Interestingly, clavicle shape is significantly different between sand and sandy soil species, and all other species (electronic supplementary material S4A), indicating that differences in habitat structure can result in changes in skeletal morphology, perhaps reflecting functional adaptation to locomotion in said environments, as already shown in studies of body shape variation [10,32,37].

Biomechanically, it remains unclear how these morphologies translate into a locomotory advantage. While they do not contact limb bones directly, clavicles are one of the main paired pectoral bones, a rigid scaffolding for muscles and connective tissue comprising the breast–shoulder apparatus, preventing the displacement of the primary girdle [11,54]. In amphisbaenians, loss of limb bones results in a restructuring and co-option of muscles previously associated with limbs to reinforce the frontal part of the body for head-first substrate penetration [4]. Similarly, the medially directed, keel-shaped clavicle we find in skinks missing forelimb bones (but not hindlimbs) may act as an anchor for the attachment of these muscles, helping to reinforce the wave of locomotion by attaching to the body wall instead of the limbs, or, as suggested by Essex [55], to reduce mechanical hindrance of muscles for burrowing and lateral undulation. As with limb proportions, the demands of undulatory movement in a granular fluid (i.e. sand) may determine similar evolutionary outcomes in skeletal morphology.

The correlation between clavicular shape and a specific stage of loss of limb elements (table 1) indicates an influence of developmental factors in generating the observed patterns. Primarily, these may be the common result of crossing a threshold of complete forelimb degeneration [56]. Given the distinction in morphospace occupation between species lacking forelimb elements and species showing vestigial limb elements, the complete truncation of the processes determining limb bones (i.e. the downregulation of the feedback mechanisms of *Hox* and *Shh* gene expression: [57–59]) may trigger consistent changes in clavicle development across clades due to shared ontogenetic pathways. As indicated by Hugi *et al*. [60], this would likely be due to a combination of shifts both in timing and speed of development (heterochrony), and a relaxation of biomechanical constraints, as the musculoskeletal links between girdles and limbs loosen and then disappear. The fact that in our study these patterns are found exclusively within a single genus (*Lerista*) may indicate that the developmental pathways involved in apparent morphological convergence are phylogenetically conserved [61], leading to similar responses to the complete loss of limb elements. Examining these developmental mechanisms across clades may help elucidate whether these mechanisms are actually convergent.

The above considerations deliberately exclude externally limbless forms, three of which we included in our analyses. Studies on girdle morphology in limbless squamates [4,18,22,23] indicate that the external loss of all limbs is a poor predictor of the structure of either girdle, pointing to a variety of morphological outcomes. This is likely due to the diminished selective pressures from locomotion acting on them [13] and stemming from divergence among taxa [9,50]. In our sample, clavicle shapes of externally limbless skinks lacking forelimb elements (*Anomalopus swansoni*, *Lerista apoda*) share close coordinates on PC1, showing some similarity (obtuse angles of the primary curvature of the clavicle) despite belonging to distinct genera (figure 1). The loss of all pectoral bones except the scapulocoracoid in *Lerista apoda* (figure 1), and their simplification in *Anomalopus swansoni*, indicate a complete loss of girdle function. Another limbless outlier, *Coeranoscincus frontalis*, retains vestigial elements of the humerus and the radio-ulna complex internally (previously undocumented, a case of cryptomelia, limbs hidden within the body wall: [16]), but its pectoral girdle is simplified and distinct from other skinks, lacking interclavicles or medial contact between clavicles (figure 1).

### (b) Limb-bearing elements as proxies of limb reduction trends

As with the clavicle, scapulocoracoid and pelvic shapes correlate with several aspects of body morphology, including the development of the respective limb pair (FE and HE), and relative limb lengths (table 1). Unlike the clavicle, the shape of pectoral and pelvic limb-bearing elements (LBEs) correlate strongly with limb reduction status (figures 2*a,b* and 3). Scapulocoracoid and pelvic shapes are ordered along the spectrum of limb reduction. When both pairs of limbs are reduced, LBEs change accordingly (table 1). This differs from clavicle shape, where body morphology and limb proportions have little effect as long as the threshold of complete degeneration of forelimb elements is not crossed. This points towards a different set of selective pressures operating on LBEs compared with non-LBEs such as the clavicle. Over half of the variation (52.3%) in pelvic shape is captured by PC1, which correlates with an increase in the angle between the two symmetrical halves of the pelvis, resulting in an antero-posteriorly shorter pelvis and an increase in the inter-ilial distance (figure 2*b*). Our findings show that species tending towards this pelvic morphology exhibit a greater degree of limb reduction. Likewise, limb reduction coincides with a simplification of scapulocoracoid shape marked by a decrease in concavity of the scapulocoracoid margin (PC1), the disappearance of the glenoid fossa (PC1 and PC2) and a straighter lateral profile (PC2) (figure 2*a*).

We find that the shape of pectoral and pelvic LBEs does not strongly associate with any specific arrangements of limb proportions, and no correlation is found with substrate ecology that would indicate functional interactions (table 1); this can be explained as a case of vestigialization, or relaxed selection following a reduction and loss of functionality [62]. As limb reduction becomes more prevalent with a shift from limbed locomotion to axial undulation [12], the selective pressures acting on LBEs will become more relaxed. In the pelvis, as already shown by studies on limb-reduced skinks [22,23,63], we observed that the iliosacral joint becomes looser and more disjointed as reduction progresses (figure 2*b*), leaving the pelvis ‘floating’ within the body wall. Due to the axial system functionally supplanting the appendicular system in the transition to undulating locomotion [12,64,65], forces transmitted by the limbs to the vertebral column in limbed locomotion become less relevant, leading to a reduction of muscle attachments, and eventually the disarticulation of the symmetrical halves of the girdle [22]. While our landmark protocol was unable to capture pelvic shape in limbless species due to its simplification, their appearance as sticks floating in the body cavity illustrates the endpoint of this process. Similarly, as limb reduction progresses, the structural need for muscle attachment diminishes, and the lateral margin of the scapulocoracoid becomes simpler and straighter (figure 2*a*).

### (c) Integration between girdle elements and influences of phylogenetic history

We find evidence of morphological integration between the pectoral and the pelvic regions in our sample of limb-reduced lizards and their limbed relatives. This applies both to the clavicle and scapulocoracoid, with the clavicle showing a stronger signal. This integration, defined as the extent to which two traits (here, the shapes of girdle elements) covary [66], is significant both in phylogenetically naive (figure 3*a*) and phylogenetically informed (figure 3*b*) tests, albeit weaker in the second. Although the anterior and posterior appendicular regions are both determined by the same ontogenetic mechanisms at a gross scale (i.e. *Hox*/*SHH* gene expression [58]), anatomical and developmental evidence suggests they represent distinct musculoskeletal modules with independent origins, excluding their serial homology [67–70]. Therefore, anatomical and developmental correlations between the two girdles should be largely explained by similar selective pressures acting upon them (e.g. locomotory interactions with the environment) or by pleiotropic effects.

One prominent candidate for such a unifying transition from limbed to limb-reduced forms is the transition from limbed to undulating locomotion [2,12,31]. The reduction of limbs may be expressed equally between the pectoral and pelvic systems (e.g. *Hemiergis*: [71]) but this is not the case in all lineages of limb-reduced squamates; otherwise, lineages reducing their forelimbs faster than the hindlimbs (i.e. *Lerista*: [2]), and *vice versa* (i.e. *Anomalopus*: [23]) would not arise. Phylogenetically naive integration plots between clavicle and pelvis (figure 3*a*) show that some correlation between the morphologies of the two girdles tends to persist as limb reduction progresses. However, species missing all elements of the forelimbs and maintaining their hindlimbs tend to be placed further from the centre of the distribution, indicating a reduced correlation between pectoral and pelvic morphology (figure 3*a*). This appears to reinforce the idea that the complete loss of one of the limb pairs is an adaptive outcome of a unique set of selective pressures (e.g. the challenges of locomoting within a granular fluid: [51]) and has profound implications in terms of both external and skeletal morphology. The finding that scapulocoracoid shape is less integrated with pelvic shape than clavicle shape may indicate that this diminishing correlation is more pronounced in LBEs compared with non-LBEs, perhaps due to the immediate changes that follow differential variations in the degrees of limb reduction each region is subjected to.

We did not observe clear patterns when considering phylogenetically controlled integration between the clavicle and pelvis (figure 3*b*). While we still obtain a gradient from fully limbed to limb-reduced species, shape correlation between the two elements is weaker and the dispersion around the regression line is higher for all taxa, and species lacking forelimb bones have more overlap with the other classes of forelimb expression (figure 3*b*). This may be because all the previously identified patterns appear consistently within selected clades (*Lerista*), weakening these relationships when relatedness is considered. For pectoral and the pelvic girdles, similar to findings in other skeletal regions (i.e. the skull: [72]), we found moderate to high phylogenetic signal (0.61 for the clavicle, 0.45 for the scapulocoracoid, 0.73 for the pelvis), indicating that a proportion of the correlated shape variation in girdles is constrained by phylogeny. For both girdle shape and limb reduction, while there may be convergent evolutionary shifts within clades, differences among clades may instead be the result of historically contingent trajectories dictated by independent evolutionary and developmental processes and selective pressures [9,10].

Despite being part of the same domain, the clavicle and scapulocoracoid are less integrated compared with either of their integration with the pelvis, and not significantly integrated when phylogeny is considered. This shows that the processes involved in the development of limb-bearing and non-limb-bearing elements within the pectoral domain are distinct, regardless of external factors such as body morphology or ecology. Perhaps, as with the integration between limb elements in mammals [73], this decoupling may also suggest distinctions in the functional morphospace occupation across taxa. The weakness of these and other shape correlations in our study may also stem from the limitations of our landmarking scheme for the clavicle and the scapulocoracoid, which due to the nature of our dataset were defined as a single landmark curve per element.

## 5. Conclusions

We examined how girdle morphology varies as limb reduction progresses in a clade of skinks. While both girdles show expected alterations in shape according to the reduction of the limbs, the way these changes are expressed shows distinct evolutionary trends. For the clavicle, across at least three lineages, the complete loss of forelimb bones when hindlimbs are retained correlates with specific clavicle morphologies, indicating either an adaptation to particular functional selective pressures, or a common developmental effect of this loss. The shape of the pelvic girdle follows trends of limb reduction more linearly, proving to be slightly better predictors of limb reduction status (proportions of limbs, digit numbers, trunk elongation), their shape change being explained by a gradual loss of functionality and biomechanical linkages with the spine as limb reduction becomes more pronounced. Integration tests between the two regions show that the shapes of the two girdles correlate, likely due to the selective pressures acting on both, but divergent outcomes can exist due to these pressures acting unequally on either girdle. However, some of this morphological variation appears to be explained by phylogenetic history.

Our study shows that, while still constrained by phylogenetic history, morphological specialization and convergence can arise in skeletal as much as external morphology, and it can mirror patterns of external morphological change as a functional response to selective pressures from the environment. It also shows how, in the context of large-scale evolutionary transformations, even minute evolutionary and developmental changes (i.e. the loss of a vestigial limb bone) can have cascading consequences on the morphology of skeletal elements across different lineages (i.e. specific clavicle shapes). Future investigations should test the functional and environmental constraints of limb-reduced morphologies while considering skeletal morphology. While several studies have compared the functional performance of various body shapes under different environmental constraints [30,74,75], none have examined the relationship between locomotion and skeletal morphology, or their biomechanical implications. Comparing locomotory performances of taxa with similar external morphology and habitat, but distinct skeletal morphologies, may elucidate the extent of which these changes are adaptive, or due to downstream ontogenetic effects.

## Data Availability

Datasets and code can be accessed at Dryad [76]. The relevant code for reproducing the analyses is hosted by Zenodo [77]. The treefile for the phylogenetic tree can be accessed at [45]. Supplementary material is available online [78].

## References

[1] Stokely PS . 1947 Limblessness and correlated changes in the girdles of a comparative morphological series of lizards. Am. Midl. Nat. **38** , 725. (10.2307/2421690)

[2] Greer AE . 1991 Limb reduction in squamates: identification of the lineages and discussion of the trends. J. Herpetol. **25** , 166. (10.2307/1564644)

[3] Jerez A , Tarazona OA . 2009 Appendicular skeleton in Bachia bicolor (Squamata: Gymnophthalmidae): osteology, limb reduction and postnatal skeletal ontogeny. Acta Zool. **90** , 42–50. (10.1111/j.1463-6395.2008.00331.x)

[4] Westphal N , Mahlow K , Head JJ , Müller J . 2019 Pectoral myology of limb-reduced worm lizards (Squamata, Amphisbaenia) suggests decoupling of the musculoskeletal system during the evolution of body elongation. BMC Evol. Biol. **19** , 16. (10.1186/s12862-018-1303-1) 30630409 PMC6329177

[5] Camaiti M , Evans AR , Hipsley CA , Chapple DG . 2021 A farewell to arms and legs: a review of limb reduction in squamates. Biol. Rev. **96** , 1035–1050. (10.1111/brv.12690) 33538028

[6] Wiens JJ , Slingluff JL . 2001 How lizards turn into snakes: a phylogenetic analysis of body-form evolution in Anguid lizards. Evolution. **55** , 2303–2318. (10.1111/j.0014-3820.2001.tb00744.x) 11794789

[7] Wiens JJ , Brandley MC , Reeder TW . 2006 Why does a trait evolve multiple times within a clade? Repeated evolution of snakelike body form in squamate reptiles. Evolution **60** , 123. (10.1554/05-328.1) 16568638

[8] Brandley MC , Huelsenbeck JP , Wiens JJ . 2008 Rates and patterns in the evolution of snake-like body form in squamate reptiles: evidence for repeated re-evolution of lost digits and long-term persistence of intermediate body forms. Evolution. **62** , 2042–2064. (10.1111/j.1558-5646.2008.00430.x) 18507743

[9] Bergmann PJ , Morinaga G . 2019 The convergent evolution of snake-like forms by divergent evolutionary pathways in squamate reptiles. Evolution. **73** , 481–496. (10.1111/evo.13651) 30460998

[10] Camaiti M , Evans AR , Hipsley CA , Hutchinson MN , Meiri S , de Oliveira Anderson R , Slavenko A , Chapple DG . 2023 Macroecological and biogeographical patterns of limb reduction in the world’s skinks. J. Biogeogr. **50** , 428–440. (10.1111/jbi.14547)

[11] Russell AP , Bauer AM . 2008 The appendicular locomotor apparatus of sphenodon and normal-limbed squamates. In The skull and appendicular locomotor apparatus of Lepidosauria, contributions to herpetology 24 (eds C Gans , AS Gaunt , K Adler ), pp. 1–465, vol. 21. Ithaca, NY: Society for the Study of Reptiles & Amphibians.

[12] Gans C . 1975 Tetrapod limblessness: evolution and functional corollaries. Am. Zool. **15** , 455–467. (10.1093/icb/15.2.455)

[13] Leonard CJ . 1979 A functional morphological study of limb regression in some southern African species of scincidae (Reptilia: Sauria). PhD dissertation, University of Cape Town, South Africa.

[14] Berger-Dell’mour HAE . 1985 The lizard genus *Tetradactylus*: a model case of an evolutionary process. In Proceedings of the international symposium of African vertebrates (ed K-L Schuchmann), pp. 495-510. Bonn: Zoologisches Forschungsinstitut und Museum Alexander Koenig.

[15] Roberts L , Head J . 2024 Independent origins of vertebral complexity in tetrapods. (10.21203/rs.3.rs-4508905/v1)

[16] Smith-Paredes D , Griffith O , Fabbri M , Yohe L , Blackburn DG , Siler CD , Bhullar BAS , Wagner GP . 2021 Hidden limbs in the “limbless skink” Brachymeles lukbani: developmental observations. J. Anat. **239** , 693–703. (10.1111/joa.13447) 33870497 PMC8349411

[17] Kearney M . 2002 Appendicular skeleton in amphisbaenians (Reptilia: Squamata). Copeia **2002** , 719–738. (10.1643/0045-8511(2002)002[0719:ASIARS]2.0.CO;2)

[18] Tsuihiji T , Kearney M , Rieppel O . 2006 First report of a pectoral girdle muscle in snakes, with comments on the snake cervico-dorsal boundary. Copeia **2006** , 206–215. (10.1643/0045-8511(2006)6[206:FROAPG]2.0.CO;2)

[19] Palci A , Caldwell MW , Scanlon JD . 2014 First report of a pelvic girdle in the fossil snake Wonambi naracoortensis Smith, 1976, and a revised diagnosis for the genus. J. Vertebr. Paleontol. **34** , 965–969. (10.1080/02724634.2014.838572)

[20] Greer AE . 1970 A subfamilial classifications of scincid lizards. Bull. Mus. Comp. Zool. **139** , 151–184.

[21] Koeller KLM . 2018 Investigating the patterns of convergence in pectoral girdle reduction during the evolution of limblessness in Lerista (Scincidae). MSc dissertation, Virginia Tech, Blacksburg, USA.

[22] Camaiti M , Villa A , Wencker LC , Bauer AM , Stanley EL , Delfino M . 2019 Descriptive osteology and patterns of limb loss of the European limbless skink Ophiomorus punctatissimus (Squamata, scincidae). J. Anat. **235** , 313–345. (10.1111/joa.13017) 31125128 PMC6637703

[23] Hutchinson MN , Couper P , Amey A , Wilmer JW . 2021 Diversity and systematics of limbless skinks (Anomalopus) from eastern Australia and the skeletal changes that accompany the substrate swimming body form. J. Herpetol. **55** , 361–384. (10.1670/20-137)

[24] Stephenson NG . 1962 The comparative morphology of the head skeleton, girdles and hind limbs in the Pygopodidae. Zool. J. Linn. Soc. **44** , 627–644. (10.1111/j.1096-3642.1962.tb01628.x)

[25] Presch W . 1975 The evolution of limb reduction in Teiid lizard genus Bachia. Bull. S. Calif. Acad. Sci. **74** , 113–121. (10.3160/0038-3872-74.3.113)

[26] Holovacs NT , Daza JD , Guerra C , Stanley EL , Montero R . 2020 You can’t run, but you can hide: the skeleton of the sand‐swimmer lizard Calyptommatus leiolepis (Squamata: Gymnophthalmidae). Anat. Rec. **303** , 1305–1326. (10.1002/ar.24246) 31469501

[27] Tran TM . 2022 Degrees of pectoral reduction on the gymnophtalmidae lizards. BSc dissertation, University of Texas at Arlington, USA.

[28] Stayton CT . 2006 Testing hypotheses of convergence with multivariate data: morphological and functional convergence among herbivorous lizards. Evolution. **60** , 824–841. 16739463

[29] Collar DC , Reece JS , Alfaro ME , Wainwright PC , Mehta RS . 2014 Imperfect morphological convergence: variable changes in cranial structures underlie transitions to durophagy in moray eels. Am. Nat. **183** , E168–E184. (10.1086/675810) 24823828

[30] Grizante MB , Brandt R , Kohlsdorf T . 2012 Evolution of body elongation in Gymnophthalmid lizards: relationships with climate. PLoS One **7** , e49772. (10.1371/journal.pone.0049772) 23166767 PMC3498171

[31] Morinaga G , Bergmann PJ . 2020 Evolution of fossorial locomotion in the transition from tetrapod to snake-like in lizards. Proc. R. Soc. B **287** , 20200192. (10.1098/rspb.2020.0192) PMC7126036 32183623

[32] Ríos-Orjuela JC , Camacho-Bastidas JS , Jerez A . 2020 Appendicular morphology and locomotor performance of two morphotypes of continental anoles: Anolis heterodermus and Anolis tolimensis. J. Anat. **236** , 252–273. (10.1111/joa.13092) 31724173 PMC6956434

[33] Tinius A , Russell AP . 2014 Geometric morphometric analysis of the breast‐shoulder apparatus of lizards: a test case using Jamaican anoles (Squamata: Dactyloidae). Anat. Rec. **297** , 410–432. (10.1002/ar.22869) 24482396

[34] Tinius A , Russell AP , Jamniczky HA , Anderson JS . 2018 What is bred in the bone: ecomorphological associations of pelvic girdle form in greater Antillean Anolis lizards. J. Morphol. **279** , 1016–1030. (10.1002/jmor.20822) 29892985

[35] Tinius A , Russell AP , Jamniczky HA , Anderson JS . 2020 Ecomorphological associations of scapulocoracoid form in greater Antillean Anolis lizards. Ann. Anat. - Anat. Anz. **231** , 151527. (10.1016/j.aanat.2020.151527) 32380193

[36] Feiner N , Jackson ISC , Stanley EL , Uller T . 2021 Evolution of the locomotor skeleton in Anolis lizards reflects the interplay between ecological opportunity and phylogenetic inertia. Nat. Commun. **12** , 1525. (10.1038/s41467-021-21757-5) 33750763 PMC7943571

[37] Camaiti M , Wiles J , Aguilar R , Hutchinson MN , Hipsley CA , Chapple DG , Evans AR . 2023 Ecomorphological correlates of inner ear shape in Australian limb-reduced skinks (Scincidae: Sphenomorphini). Zool. J. Linn. Soc. **199** , 994–1012. (10.1093/zoolinnean/zlad074)

[38] Greer AE . 1989 The biology and evolution of Australian lizards. Chipping Norton, Australia: Surrey Beatty and Sons.

[39] Shea GM . 2021 Nomenclature of supra-generic units within the family scincidae (squamata). Zootaxa **5067** , 301–351. (10.11646/zootaxa.5067.3.1) 34810739

[40] Slavenko A , Dror L , Camaiti M , Farquhar JE , Shea GM , Chapple DG , Meiri S . 2022 Evolution of diel activity patterns in skinks (squamata: scincidae), the world’s second-largest family of terrestrial vertebrates. Evolution. **76** , 1195–1208. (10.1111/evo.14482) 35355258 PMC9322454

[41] Chapple DG , Slavenko A , Tingley R , Farquhar JE , Camaiti M , Roll U , Meiri S . 2023 Built for success: distribution, morphology, ecology and life history of the world’s skinks. Ecol. Evol. **13** , e10791. (10.1002/ece3.10791) 38094152 PMC10716605

[42] Camaiti M , Evans AR , Hipsley CA , Hutchinson MN , Meiri S , Anderson RO , Slavenko A , Chapple DG . 2022 A database of the morphology, ecology and literature of the world’s limb‐reduced skinks. J. Biogeogr. **49** , 1397–1406. (10.1111/jbi.14392)

[43] Pollock TI , Hocking DP , Evans AR . 2022 The killer’s toolkit: remarkable adaptations in the canine teeth of mammalian carnivores. Zool. J. Linn. Soc. **196** , 1138–1155. (10.1093/zoolinnean/zlab064)

[44] Adams DC , Collyer ML , Kaliontzopoulou A , Baken EK . 2022 Geomorph: Software for geometric morphometric analyses. R package version 4.0.3. See https://cran.r-project.org/package=geomorph.

[45] Zheng Y , Wiens JJ . 2016 Combining phylogenomic and supermatrix approaches, and a time-calibrated phylogeny for squamate reptiles (lizards and snakes) based on 52 genes and 4162 species. Mol. Phylogenet. Evol. **94** , 537–547. (10.1016/j.ympev.2015.10.009) 26475614

[46] Pennell MW , Eastman JM , Slater GJ , Brown JW , Uyeda JC , FitzJohn RG , Alfaro ME , Harmon LJ . 2014 Geiger v2.0: an expanded suite of methods for fitting macroevolutionary models to phylogenetic trees. Bioinformatics **30** , 2216–2218. (10.1093/bioinformatics/btu181) 24728855

[47] Adams DC . 2014 A generalized K statistic for estimating phylogenetic signal from shape and other high-dimensional multivariate data. Syst. Biol. **63** , 685–697. (10.1093/sysbio/syu030) 24789073

[48] Revell LJ . 2012 Phytools: an R package for phylogenetic comparative biology (and other things). Methods Ecol. Evol. **3** , 217–223. (10.1111/j.2041-210X.2011.00169.x)

[49] Collyer ML , Adams DR . 2019 Linear model evaluation with randomized residuals in a permutation procedure. R package version 0.4. See https://cran. r-project. org/package= RRPP.

[50] Morinaga G , Bergmann PJ . 2017 Convergent body shapes have evolved via deterministic and historically contingent pathways in Lerista lizards. Biol. J. Linnean Soc. **121** , 858–875. (10.1093/biolinnean/blx040)

[51] Pough FH , Preest MR , Fusari MH . 1997 Prey-handling and the evolutionary ecology of sand-swimming lizards (Lerista: Scincidae). Oecologia **112** , 351–361. (10.1007/s004420050320) 28307484

[52] Attum O , Eason P , Cobbs G . 2007 Morphology, niche segregation, and escape tactics in a sand dune lizard community. J. Arid Environ. **68** , 564–573. (10.1016/j.jaridenv.2006.07.010)

[53] Crofts SB , Summers AP . 2011 Swimming in the Sahara. Nature **472** , 177–178. (10.1038/472177a) 21490665

[54] Peterson JA . 1973 Adaptation for arboreal locomotion in the shoulder region of lizards. PhD dissertation, The University of Chicago, USA.

[55] Essex R . Studies in reptilian degeneration. In Proceedings of the Zoological Society of London, pp. 879–945. Oxford, UK: Blackwell Publishing Ltd.

[56] Woltering JM . 2012 From lizard to snake; behind the evolution of an extreme body plan. Curr. Genomics **13** , 289–299. (10.2174/138920212800793302) 23204918 PMC3394116

[57] Shapiro MD , Hanken J , Rosenthal N . 2003 Developmental basis of evolutionary digit loss in the Australian lizard Hemiergis. J. Exp. Zool. B Mol. Dev. Evol. **297** , 48–56. (10.1002/jez.b.19) 12955843

[58] Hinchliffe JR . 2002 Developmental basis of limb evolution. Int. J. Dev. Biol. **46** , 835–845. (10.1387/ijdb.12455618) 12455618

[59] Leal F , Cohn MJ . 2018 Developmental, genetic, and genomic insights into the evolutionary loss of limbs in snakes. Genesis **56** , e23077. (10.1002/dvg.23077) 29095557

[60] Hugi J , Hutchinson MN , Koyabu D , Sánchez-Villagra MR . 2012 Heterochronic shifts in the ossification sequences of surface- and subsurface-dwelling skinks are correlated with the degree of limb reduction. Zoology **115** , 188–198. (10.1016/j.zool.2011.10.003) 22502802

[61] Kopp A . 2009 Metamodels and phylogenetic replication: a systematic approach to the evolution of developmental pathways. Evolution. **63** , 2771–2789. (10.1111/j.1558-5646.2009.00761.x) 19545263

[62] Fong DW , Kane TC , Culver DC . 1995 Vestigialization and loss of nonfunctional characters. Annu. Rev. Ecol. Syst. **26** , 249–268. (10.1146/annurev.es.26.110195.001341)

[63] Miralles A , Hipsley CA , Erens J , Gehara M , Rakotoarison A , Glaw F , Müller J , Vences M . 2015 Distinct patterns of desynchronized limb regression in Malagasy scincine lizards (Squamata, Scincidae). PLoS One **10** , e0126074. (10.1371/journal.pone.0126074) 26042667 PMC4456255

[64] Chong B , Wang T , Erickson E , Bergmann PJ , Goldman DI . 2022 Coordinating tiny limbs and long bodies: geometric mechanics of lizard terrestrial swimming. Proc. Natl Acad. Sci. USA **119** , e2118456119. (10.1073/pnas.2118456119) 35759665 PMC9271186

[65] Lande R . 1978 Evolutionary mechanisms of limb loss in tetrapods. Evolution. **32** , 73–92. (10.1111/j.1558-5646.1978.tb01099.x) 28564089

[66] Klingenberg CP . 2014 Studying morphological integration and modularity at multiple levels: concepts and analysis. Phil. Trans. R. Soc. B **369** , 20130249. (10.1098/rstb.2013.0249) 25002695 PMC4084535

[67] Sears KE , Capellini TD , Diogo R . 2015 On the serial homology of the pectoral and pelvic girdles of tetrapods. Evolution. **69** , 2543–2555. (10.1111/evo.12773) 26374500

[68] Burke AC . 1991 Proximal elements in the vertebrate limb: evolutionary and developmental origin of the pectoral girdle. In Developmental patterning of the vertebrate limb (eds JR Hinchliffe , JM Hurle , D Summerbell ), pp. 385–394. New York, NY: Springer Science & Business Media, Plenum Press. (10.1007/978-1-4615-3310-8_49)

[69] Zhu M , Yu X , Choo B , Wang J , Jia L . 2012 An antiarch placoderm shows that pelvic girdles arose at the root of jawed vertebrates. Biol. Lett. **8** , 453–456. (10.1098/rsbl.2011.1033) 22219394 PMC3367742

[70] Soliz MC , Ponssa ML , Abdala V . 2018 Comparative anatomy and development of pectoral and pelvic girdles in Hylid anurans. J. Morphol. **279** , 904–924. (10.1002/jmor.20820) 29665044

[71] Choquenot D , Greer AE . 1989 Intrapopulational and interspecific variation in digital limb bones and presacral vertebrae of the genus Hemiergis (Lacertilia, Scincidae). J. Herpetol. **23** , 274. (10.2307/1564449)

[72] Stepanova N , Bauer AM . 2021 Phylogenetic history influences convergence for a specialized ecology: comparative skull morphology of African burrowing skinks (Squamata; Scincidae). BMC Ecol. Evol. **21** , 86. (10.1186/s12862-021-01821-w) 33993867 PMC8127277

[73] Richards HL , Rovinsky DS , Adams JW , Evans AR . 2023 Inferring the palaeobiology of palorchestid marsupials through analysis of mammalian humeral and femoral shape. J. Mammal. Evol. **30** , 47–66. (10.1007/s10914-022-09640-6)

[74] Bergmann PJ , Irschick DJ . 2010 Alternate pathways of body shape evolution translate into common patterns of locomotor evolution in two clades of lizards. Evolution. **64** , 1569–1582. (10.1111/j.1558-5646.2009.00935.x) 20050911

[75] Gans C , Fusari M . 1994 Locomotor analysis of surface propulsion by three species of reduced‐limbed fossorial lizards (Lerista: Scincidae) from western Australia. J. Morphol. **222** , 309–326. (10.1002/jmor.1052220308) 29865419

[76] Camaiti M , Hutchinson MN , Hipsley CA , Aguilar R , Black J , Chapple DG , Evans AR . 2024 Patterns of girdle shape and their correlates in Australian limb-reduced skinks. Dryad Digital Repository. (10.5061/dryad.mw6m9065d)39353558

[77] Camaiti M , Hutchinson M , Hipsley C , Aguilar R , Black J , Chapple D , Evans A . 2024 Patterns of girdle shape and their correlates in Australian limb-reduced skinks. Zenodo. (10.5281/zenodo.13306797)39353558

[78] Camaiti M , Hutchinson M , Hipsley CA , Aguilar R , Black JR , Chapple DG *et al* . 2024 Data from: Patterns of girdle shape and their correlates in Australian limb-reduced skinks. Figshare. (10.6084/m9.figshare.c.7468085)39353558

